# Non-Invasive Brain-to-Brain Interface (BBI): Establishing Functional Links between Two Brains

**DOI:** 10.1371/journal.pone.0060410

**Published:** 2013-04-03

**Authors:** Seung-Schik Yoo, Hyungmin Kim, Emmanuel Filandrianos, Seyed Javid Taghados, Shinsuk Park

**Affiliations:** 1 Department of Radiology, Brigham and Women’s Hospital, Harvard Medical School, Boston, Massachusetts, United States of America; 2 Department of Mechanical Engineering, Korea University, Seoul, Korea; 3 Department of Biomedical Engineering, Boston University, Boston, Massachusetts, United States of America; 4 School of Nano-Bioscience and Chemical Engineering, Ulsan National Institute of Science and Technology, Ulsan, Korea; 5 Center for Bionics, Korea Institute of Science and Technology, Seoul, Korea; Hosptial Infantil Universitario Niño Jesús, CIBEROBN, Spain

## Abstract

Transcranial focused ultrasound (FUS) is capable of modulating the neural activity of specific brain regions, with a potential role as a non-invasive computer-to-brain interface (CBI). In conjunction with the use of brain-to-computer interface (BCI) techniques that translate brain function to generate computer commands, we investigated the feasibility of using the FUS-based CBI to non-invasively establish a functional link between the brains of different species (*i.e.* human and **Sprague-Dawley** rat), thus creating a brain-to-brain interface (BBI). The implementation was aimed to non-invasively translate the human volunteer’s intention to stimulate a rat’s brain motor area that is responsible for the tail movement. The volunteer initiated the intention by looking at a strobe light flicker on a computer display, and the degree of synchronization in the electroencephalographic steady-state-visual-evoked-potentials (SSVEP) with respect to the strobe frequency was analyzed using a computer. Increased signal amplitude in the SSVEP, indicating the volunteer’s intention, triggered the delivery of a burst-mode FUS (350 kHz ultrasound frequency, tone burst duration of 0.5 ms, pulse repetition frequency of 1 kHz, given for 300 msec duration) to excite the motor area of an anesthetized rat transcranially. The successful excitation subsequently elicited the tail movement, which was detected by a motion sensor. The interface was achieved at 94.0±3.0% accuracy, with a time delay of 1.59±1.07 sec from the thought-initiation to the creation of the tail movement. Our results demonstrate the feasibility of a computer-mediated BBI that links central neural functions between two biological entities, which may confer unexplored opportunities in the study of neuroscience with potential implications for therapeutic applications.

## Introduction

Brain-to-computer interface (BCI) refers to the hardware and software environment that detects and translates brain activity to control computers or stored-program architecture devices without involving muscles or the peripheral nervous system [1]. To characterize a specific function of the brain, invasive means such as implantable cortical microelectrode arrays that directly detect the electrical field potentials/spikes from the somatomotor areas have been used, for example, to provide BCI control options for quadriplegic patients [2]. Nicolelis and colleagues explored the method of obtaining the neural electrical signals directly from the motor cortex of primates using an implanted cortical electrode array, and decoded the signals obtained during complex motor intentions, into the appropriate machine control [3]. Velliste *et al.* used intracortical recording schemes in monkeys to convert motor cortex neural activity into a correlated mechanized prosthetic arm movement used for self-feeding [4]. Other than these BCI methods which require a surgery to implant electrodes to the brain surface, non-invasive functional imaging modalities such as electroencephalogram (EEG) and functional magnetic resonance imaging (fMRI) have also been adopted in implementation of BCI. For example, non-invasive EEG-based BCI, with the combinatory inclusion of navigation algorithms, was successfully implemented to allow for thought processes to control the direction of a wheelchair movement [5]. Yoo and colleagues used fMRI, with real-time processing capabilities, to provide computer cursor directional commands based on spatial patterns of cortical activity that were linked to predetermined thought processes [6]. This ability was later expanded to the generation of computer keyboard commands via combining spatial activation patterns with different temporal hemodynamic patterns associated with the task onset delays controlled by human subjects [7], [8]. Magneto-encephalography (MEG), near infrared spectroscopy (NIRS), and functional trascranial doppler sonography (fTCD) have also emerged recently as potential candidates for non-invasive BCI (reviewed in [9]).

It is notable that the flow of information used in the current implementation of BCI is unidirectional, in the sense that the control commands originating from the brain are directed to operate a computer. To establish the bidirectional interface between the brain and the computer, the creation of a computer-to-brain interface, namely CBI, was sought after, whereby the computer-generated commands can be used to modulate the function of the specific brain area *via* its direct stimulation/suppression, all without engaging the peripheral nervous system and sensory pathways. The bidirectional interface between the brain and the computer would ultimately lead to the development of a ‘Brain-to-Brain Interface’ (BBI), in which neural activities from individual brains are linked and mediated by computers [9].

Modern brain stimulation techniques, which typically utilize a computer/electrical circuits for operation, can potentially be used for CBI application under the presence of linkage to a computer. For example, direct electrical stimulation of the motor cortex, achieved by surgically-implanted electrodes, was used to elicit animal limb motion necessary for navigating through complex spatial environments [10]. Deep brain stimulation (DBS) or epicortical stimulation can also be adopted for human application, but would require invasive surgical procedures [11]. Transcranial magnetic stimulation (TMS) confers the non-invasive means of neuromodulation; however, lacks penetration depth and spatial specificity due to its electromagnetically inductive nature [11].

Transcranial sonication of focused ultrasound (FUS) has emerged as a new breed of non-invasive region-specific brain stimulation technique. Since the seminal feasibility study of Fry *et al.* back in the late 1950s [12], the neuromodulatory potentials of ultrasound have been demonstrated in *ex vivo* tissues [13] and more recently in rodent models [14]. Transcranial FUS techniques deliver highly focused acoustic energy to the localized deep regions of the brain, and have been used in thermal ablation of brain tumors [15] and functional neurosurgery [16]. When given in pulsed mode at low acoustic energy, far below the thermal or cavitation threshold which may damage the underlying tissue, FUS is capable of modulating the excitability of sonicated tissues. This ability has been demonstrated in excitation/suppression of rabbit motor/visual cortices [17]. Furthermore, FUS has proven itself as a versatile means of non-invasive neuromodulation in the suppression of chemically-induced epilepsy [18] and in altering the concentrations of extracellular neurotransmitters [19], [20]. Most of the current FUS devices are controlled by a computer, making them favorable candidates for the CBI.

With realization of FUS-based non-invasive neuromodulation as a CBI, we were motivated to implement a novel concept of BBI by combining the EEG-based BCI and FUS-based CBI. Using a processing computer as an interface between the two, the implementation is straightforward. A thought-process (intention to stimulate a rat brain) originating from a human participant is detected in forms of EEG-based steady-state visual evoked potential (SSVEP). Upon detection, a computer triggers the operation of the FUS that stimulates the motor cortex of a rat (Sprague-Dawley), which elicits the subsequent tail movement.

## Materials and Methods

### Overview

**Figure 1:**
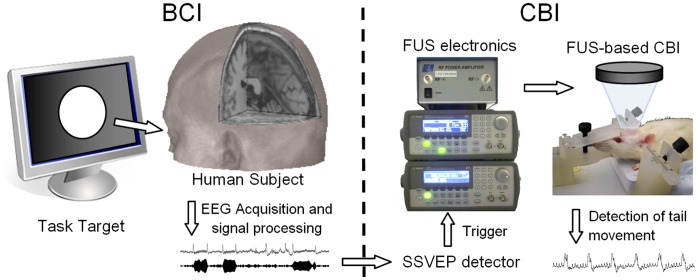
The schematics of the implemented brain-to-brain interface (BBI). The implementation consists of steady-state visual evoked potential (SSVEP)-based brain-to-computer interface (BCI: on the left column) and focused ultrasound (FUS)-based computer-to-brain interface (CBI) segments (on the right column).

This study was conducted under approval by the Partners Human Research Committee (Institutional Review Board of Brigham and Women’s Hospital, Partners Healthcare Systems) for the study involving humans, and by the Harvard Medical Area Standing Committee on Animals for the experimental portion involving animals. All experiments were conducted within the premise of Brigham and Women’s Hospital, Harvard Medical School. All the participants provided their written informed consent to participate in the study according to the approved procedures set forth by the IRB. The overall set-up of the BBI configuration is depicted in Figure 1, which consists of BCI and CBI segments. In the BCI segment, EEG signals obtained from the operator *via* single-montage surface electrodes are processed by a computer. The synchronization of the EEG signal fluctuation with respect to the external visual stimuli occurs only when the individual actively gazes at the stimulus source (thus, generating the SSVEP). This synchronization manifests itself in the form of increased signal amplitude in the EEG bandwidth corresponding to the specific visual stimulation frequency [21].

SSVEP is a widely-accepted detection mechanism used in the context of BCI. SSVEP signals are generated only when the participant intentionally gazes at the flickering light source [22], [23], and the user’s act of actively focusing on the flicker source is indispensible to the actuation of the BCI system. Due to robust responses of SSVEP across test subjects, along with high performance accuracy after only a short training period [24] or even no prior experience [23], SSVEP is considered to provide excellent alternatives to other EEG-base BCI approaches, for example, P300 component or event-related desynchronization [25]. Once detected by a computer algorithm, the SSVEP subsequently triggers the operation of the FUS-based, non-invasive brain stimulation device that stimulates the motor areas of the rat’s brain. The associated tail movement is recorded for further data analysis.

### Implementation of SSVEP-based BCI

The visual stimuli necessary for the SSVEP-based BCI were generated by Matlab codes (Mathworks, Natick, MA) using modification of the Psychophysics Toolbox extensions [26], [27]. The flickering (black and white) circle was displayed in the center of a gray background on a computer monitor at different frequencies (5, 10, 15, and 20 Hz respectively). The visual angle from the subject to monitor was maintained at approximately 10 degrees. The EEG was measured from the single montage (Fp1-O1) surface electrodes using data acquisition hardware (Dual BioAmp ML408, PowerLab 16/30, ADInstruments, CO) at a sampling rate of 1 kHz (Mains filter was applied to reduce the ambient radiofrequency noise). The acquired EEG signal was filtered through a digital band-pass filter centered at the flickering frequency (10% bandwidth, LabChart 7, ADInstruments, CO). The increased level of the resulting EEG signal amplitude, therefore, reflected the degree of synchronization of the visual neural signals with the external light stimuli.

### Parameter Optimization for the SSVEP-based BCI

To verify the feasibility of using SSVEP for the operation of the BCI and to optimize the experimental parameters for the detection accuracy (*i.e.*, a threshold level used in the signal detection algorithm and the frequency of the strobe), seven healthy human volunteers (age = 30.6±9.3 years old, two females) who do not have a history of neurological disorders were recruited. After the application of EEG electrodes, the volunteers were asked to look away from the flickering target as a baseline condition (non-gaze) without closing their eyes. Subsequently, they were instructed to gaze at the flickering patterns for duration of approximately five seconds, followed again by the baseline condition. Head movement was discouraged during the gaze/non-gaze task. The subjects repeated the task 20 times, interleaved with an equal number of non-task periods. A verbal cue (*viz.* “proceed to the next”) was given by the staff to indicate that the 25-second minimum interval between the tasks had elapsed, and that the subject was free to engage in the flickering screen *ad libitum*. No other forms of instructions were given. Each subject notified the timing of task initiation to examiners nonverbally, using a thumbs-up gesture, which was detected by a motion-sensitive probe attached to the finger (MLT 1010, ADInstruments, CO). The potential confounding effects on the acquired EEG, such as appearance of motion-related evoked potentials or motion-related artifacts, which are associated with this thumbs-up gesture, were evaluated from three individuals (age = 44.3±2.5 years old, one female). The same data EEG acquisition as well as task procedures were taken, without presenting the visual flickers.

Upon data acquisition, the standard deviation (SD) of signal fluctuations was calculated from the first 10 second-segment during the baseline, non-gaze condition. The task-related SSVEP was detected by applying a threshold of four, five, six and seven times SD of the baseline EEG signal. The occurrences of false positives (FP; task detection during baseline condition), true negatives (TN; no task detection during baseline period), true positives (TP; task detection during gazing period) and false negatives (FN; no task detection during gazing period) were calculated, and the detection accuracy was characterized using accuracy index (ACC) and F1-score (ACC = (TP+TN)/(P+N); F1 = 2 TP/(P+P’), where P = TP+FN, N = FP+TN, and P’ = TP+FP [28]).

### Implementation of FUS-based CBI

An air-backed, spherical-segment, piezoelectric ultrasound transducer (6 cm in outer diameter; 7 cm in radius-of-curvature), operating at a fundamental frequency of 350 kHz, was used to deliver focused acoustic pressure waves to the specific region-of-interest of the rat (Sprague–Dawley) brain. The acoustic field at the focus was characterized in 3-dimensional space according to the method described previously [17], and was roughly cigar-shaped and measuring 6.5 mm in diameter at the full-width-at-half maximum (FWHM) of the acoustic pressure field. The animal was anesthetized using an intraperitoneal injection of a 80 mg/kg ketamine and 10 mg/kg xylazine mixture. After shaving the rat’s scalp, the FUS transducer was coupled to the rat’s head through a plastic bag filled with degassed water. For effective transmission of acoustic energy, ultrasound gel was applied between the rodent’s scalp and the plastic bag.

The input signal to the FUS transducer was generated by two function generators (FG) (33210 A, Agilent, Santa Clara, CA) and concurrently amplified by a linear power amplifier (240 L, ENI Inc, Rochester, NY). Two FGs were used to create the pulsed operation of sonication; the first generator controlled the overall duration of sonication (300 msec) and pulse repetition frequency (PRF: 1 KHz); the second generator, triggered by the first, generated a sinusoidal waveform at 350 kHz with a tone burst duration (TBD) of 0.5 msec. The sonication parameters were selected based on our previous investigation regarding the excitatory effects of sonication in rats [17]. For the calculation of acoustic intensity, the pressure amplitude was estimated after taking into account ultrasound attenuation through the rat skull *in situ* (∼87% of the incoming sonication pressure). The corresponding spatial-peak pulse-average intensity (I_sppa_) was 8.6 W/cm^2^, and the mechanical index (MI) was 0.9 (where peak negative pressure was 0.53 MPa), where the MI is the index used to indicate the possibility of pressure-related biological tissue damage. Taking into consideration the duty factor given by the TBD and PRF (*i.e.*, 50%), the resulting spatial-peak temporal-average intensity (I_spta_) was 4.3 W/cm^2^.

According to the functional map of the rat motor cortex [29], the sonication focus was targeted to the area associated with tail movement, which is located ∼2 mm posterior to Bregma. The location of the sonication focus was confirmed using a optically-tracked image-guidance system [19]. The rat’s tail movement was detected by a motion sensor (MLT 1010, ADInstruments, CO) wrapped around the ***caudal appendage***. The motion signal was obtained at 1 kHz sampling rates using data acquisition device and software (PowerLab 16/30 and LabChart 7; ADInstruments, CO). The FUS-mediated tail motion was defined when the measured motion signal exceeded five times the SD of the signal fluctuations calculated from the first ten second-segments during the initial baseline data acquisition period. The occurrences of the tail motion, as well as the time delay between the onset of FUS-operation and the tail movement, were measured.

### Interface between BCI and CBI

According to the outcome of the parameter optimization for SSVEP-based BCI (please see the Result Section), visual stimulation of 15 Hz was used while the thresholds of 5 standard deviations (SDs) of the baseline signal fluctuation was used to detect the task-specific SSVEP. Computer software (LabChart 7, ADInstruments, CO) was programmed to automatically detect the amplitude of the SSVEP that exceeds the threshold condition, and triggered the operation of open-source electronic I/O board (Arduino, Sparkfun Electronics, Boulder, CO), which generated a square pulse (5 V, duration 10 msec) to initiate the operation of FUS-based CBI device. Trigger circuitries between BCI and CBI did not introduce any detectable time delay. Once triggered, the circuit was automatically disabled for 10 seconds to prevent repetitive operation of the CBI circuit (which may induce an excessive excitation of the brain area).

### BBI Implementation

A total of six newly-recruited, previously untrained individuals (age 42.3±8.1 years old, 2 females) participated in the BBI implementation. Six counterpart Sprague–Dawley rats (weight 363±37 g, all males) were also used. The same SSVEP-based BCI set-up and parameters employed during the optimization process were applied to detect the individual’s intention to stimulate the rat’s brain. A brief single training trial (each lasting two or three minutes to check the status of the equipment) was given to the participants prior to the BBI sessions. Each subject’s baseline EEG was measured for 10 seconds to determine the level of baseline signal fluctuation. The subject was then instructed to gaze at the computer monitor (bearing 15 Hz flicker) *ad libitum* to initiate his/her intention to stimulate the rodent’s motor cortex and elicit tail movement; the subject was also instructed to sustain their gaze for 4–5 seconds. The minimum interval between the tasks was maintained at 25 seconds. To allow experimenters to record the initiation of the task, each individual signaled their intention using thumbs-up gesture, which was detected by the motion-sensitive probe. The task-specific SSVEP triggered the subsequent operation of the FUS-based CBI, which resulted in the sonication of a rat positioned in the FUS setup under stereotactic guidance. The successful stimulation of the motor area was examined using corresponding tail movement as detected by a motion sensor. The success rates, *i.e.,* ACC and F1 scores were calculated from a total of 20 trials per each BBI experiment. Subjects were not provided feedback on whether the tail movement from the rat was successful. This was done in order to allow the subjects to engage/concentrate on the visual SSVEP task without the potential for attention/visual distractions.

## Results

### Parameter Optimization for the SSVEP-based BCI

**Figure 2:**
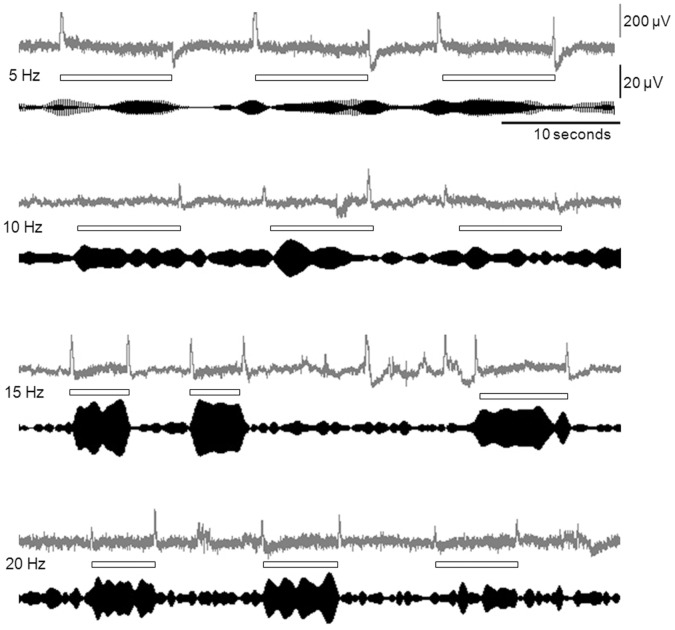
An example of raw and filtered SSVEP. A raw SSVEP (in gray lines) and the signal after the application of the digital filter at the corresponding stimulation frequency (in black lines), obtained from a volunteer from four different stimulation frequencies (5, 10, 15 and 20 Hz). The rectangular box indicates the time the operator intended to engage the task.

The example of SSVEP recordings from one subject, as well as the signals that were filtered at each visual stimulation frequency, are shown in Fig. 2. At a frequency of 5 Hz stimulation, the filtered EEG signal showed slight elevation in the signal amplitude compared to baseline. The amplitude became progressively more distinct at 10 Hz and 15 Hz; however, the signal quality became less distinct at 20 Hz, suggesting that 15 Hz appears to create EEG responses that are synchronized with the visual stimulation.

Different combinations of visual stimulation frequencies and detection thresholds were applied to all human subjects (n = 7), and subsequent detection accuracies, in terms of averaged values of FP/TN/TP/FN scores (Table 1) and AAC and F1-score, were tabulated (Table 2). The result showed that stimulation frequency of 15 Hz and detection threshold of 5 SDs of the baseline noise level (as marked in bold font) generated the highest detection accuracy (0.94 in Table 2; corresponding averaged TN score of 18.4 and TP score of 19.1–20 was the maximum score for the best accuracy, Table 1), with the average response time of 1.39±0.49 sec.

**Table 1:** Optimization of BCI parameter.

		4 SD	5 SD	6 SD	7 SD
5 Hz	FP/TN	6.7±7.5/13.0±7.3	3.1±5.0/16.7±5.0	1.0±1.7/19.0±1.7	0.3±0.8/19.7±0.8
	TP/FN	8.7±7.6/11.6±7.8	5.1±6.8/15.0±6.9	2.0±5.9/17.0±5.9	1.6±3.7/18.4±3.7
10 Hz	FP/TN	6.4±5.9/13.4±6.1	3.6±4.3/16.3±4.5	2.3±3.1/17.6±3.4	1.7±2.2/18.1±2.5
	TP/FN	19.3±1.5/0.6±1.5	18.0±3.3/1.9±3.3	16.0±5.6/3.9±5.6	13.1±6.9/6.7±6.8
15 Hz	FP/TN	3.3±3.8/16.7±3.8	**1.6**±**3.4/18.4**±**3.4**	0.7±1.9/19.3±1.9	0.4±1.1/19.6±1.1
	TP/FN	20.0±0.0/0.0±0.0	**19.1**±**1.5/0.9**±**1.5**	15.6±5.1/4.4±5.1	12.4±6.6/7.6±6.6
20 Hz	FP/TN	5.4±4.1/14.6±4.1	3.0±2.6/17.0±2.6	1.0±1.0/19.0±1.0	0.9±1.2/19.1±1.2
	TP/FN	17.6±4.4/2.4±4.4	15.1±8.0/4.9±8.0	13.1±9.1/6.9±9.1	10.7±8.7/9.3±8.7

False Positive/True Negative (FP/TN) and True Positive/False Negative (TP/FN) values in various flickering and peak detection thresholds (in terms of standard deviation (SD) of the baseline EEG signal) are presented. The values were obtained from 20 task/non-task periods averaged from seven participants (mean ± s.d.).

**Table 2:** Distribution of AAC and F1-scores across the participants (n = 7) in various flickering frequencies and peak detection thresholds in terms of standard deviation (SD) of the baseline EEG signal.

	4 SD ACC/F1	5 SD ACC/F1	6 SD ACC/F1	7 SD ACC/F1
5 Hz	0.52±0.23/0.44±0.33	0.55±0.17/0.30±0.30	0.55±0.12/0.18±0.29	0.53±0.07/0.10±0.23
10 Hz	0.82±0.15/0.86±0.11	0.86±0.12/0.87±0.11	0.84±0.14/0.81±0.23	0.79±0.16/0.71±0.28
15 Hz	0.92±0.10/0.93±0.08	**0.94±0.12/0.94±0.10**	0.87±0.17/0.84±0.22	0.80±0.18/0.71±0.29
20 Hz	0.80±011/0.81±0.13	0.80±0.17/0.73±0.31	0.80±0.21/0.66±0.45	0.75±0.21/0.57±0.43

ACC = (TP+TN)/(P+N); F1 = 2 TP/(P+P’), where P = TP+FN, N = FP+TN, and P’ = TP+FP, from Table 1.

From a separate evaluation of the EEG signal acquired using only the thumbs-up gesture (n = 3), it was found that no gesture-related signals were detected from any of the participants at the threshold conditions used in the BCI implementation, suggesting that the gesture itself did not confound the accuracy of the SSVEP-BCI.

### Implementation of BBI

**Figure 3:**
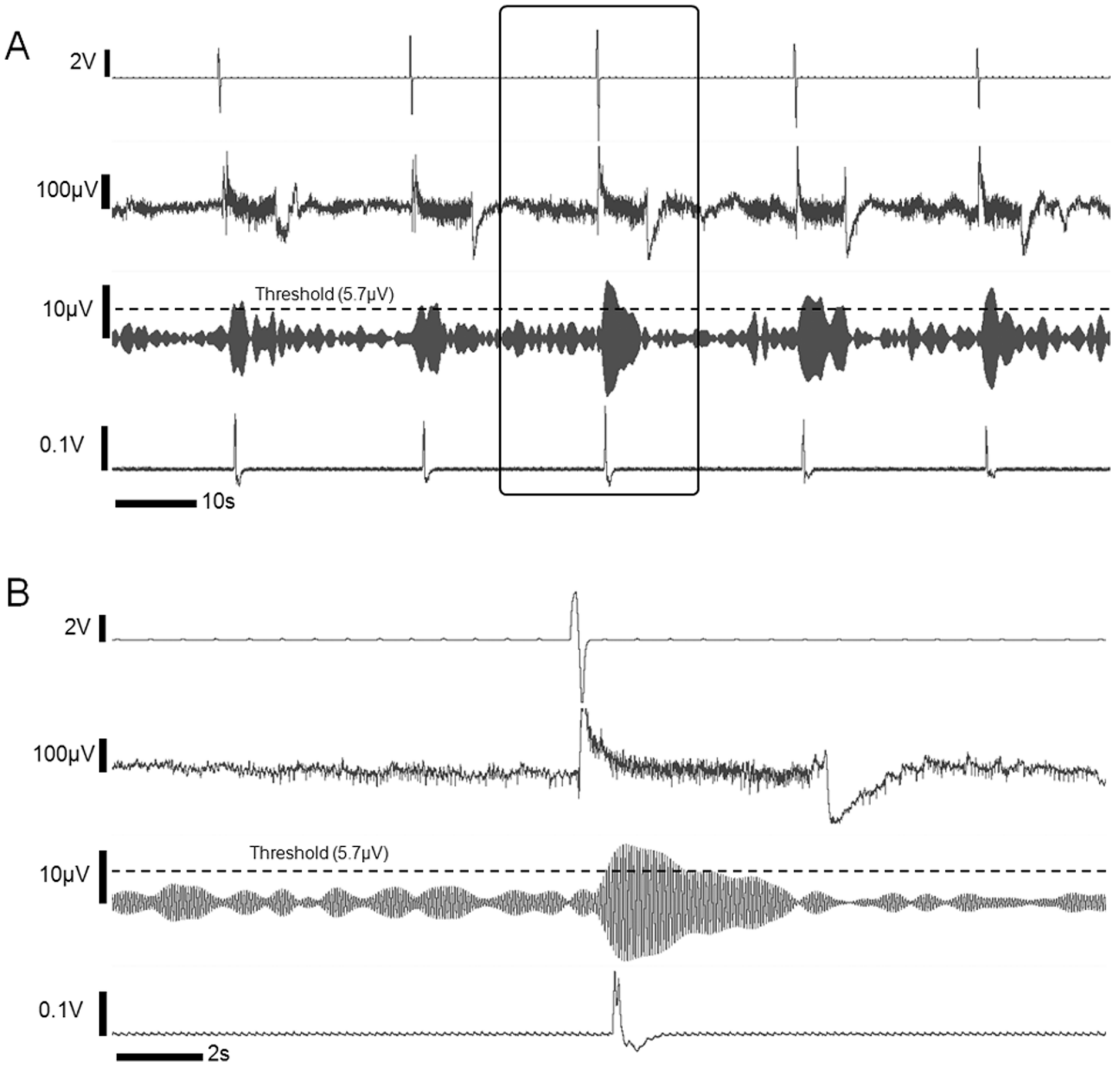
Example of bio-signals obtained from the BBI operation. (A) Initiation of operator intension (as signaled by the finger movement; top), the raw EEG data (the 2^nd^ row), the filtered EEG data at 15 Hz (the 3^rd^ row), and the detected rat tail movement (the last row). The threshold condition for the filtered EEG is shown in dotted line. (B) The time resolved plot of the box shown in (A).

All six participants were able to execute the intended BBI task. An example of the initiation of operator intention, detected raw and filtered SSVEP data, and subsequent tail motion of the rat is shown in Fig. 3 (also see Video S1). AAC and F1-scores for the BCI segment of the experiment were 0.94±0.03 and 0.94±0.04 respectively, with corresponding FP/TN/TP/FN values of 2.0±1.3/20.0±0.0/19.5±1.2/0.5±1.2 (n = 6). The average response time between user intension to the trigger and the operation of the FUS was 1.59±1.07 sec. For the CBI segment of the operation, AAC and F1-scores (*i.e*. 1.00±0.01 for both; n = 6) indicated the high rate of stimulation accuracy. All sonication trials, except one (which was false positive activation) out of 120 independent trials, resulted in successful activation of the rat motor cortex and subsequent tail movement. Considering there were no tangible delays introduced by triggering circuits and by acoustic wave propagation to the target (on the order of 47 µsec), the latency between the initiations of sonication to the detected tail movement was 0.24±0.05 sec.

## Discussion

We present a method for non-invasive functional linkage of brain activity between human volunteers and Sprague–Dawley rats. Our results demonstrate the feasibility of computer-mediated interfacing of the neural signals between human and animal to generate simple motor responses. Optimization of the stimulus frequency (15 Hz) and the detection threshold (five times standard deviations of baseline noise level) for the analysis of the bandwidth-filtered SSVEP signals attained high-degree of performance accuracy for the BCI (∼94%). These findings are congruent with the high performance accuracy of SSVEP BCI, whereby 95% average accuracy was attainable from only few runs (up to four) with the presentation of a 4-minute long visual stimulation in a group of human volunteers [24]. Although SSVEP-based BCI used in the present study has been utilized across a wide spectrum of subjects without dedicated training or optimization of the detection algorithm, the expansion of the degrees of control options, using alternative BCI modalities (both invasive and non-invasive means), will inevitably require extensive training for performance accuracy and minimal time delays since the component of learning and corresponding functional modulation/plasticity are implicated in the use of BCI [30], [31].

FUS-mediated CBI operation was also highly accurate in stimulating the rat motor cortex. The acoustic intensity used for the CBI operation was 4.3 W/cm^2^ I_spta_ with corresponding MI of 0.9. Similar sonication parameters have been applied to animal brains without causing short or long term biological damages [17]. Other studies have indicated that neural excitation could occur at even lower acoustic intensity and pressure [13] [32]. and therefore, it is reasonable to predict that lower sonication intensity may also be used as CBI for human application. The time delay between the initiation of user-intention and the triggering of FUS apparatus was on the order of a few seconds (1.59 sec), which is typical time for the EEG signals to reach steady-state upon the initiation of the task. Adoption of a different EEG-based BCI technique with a faster response, such as non-steady-state evoked potential acquisition, would reduce the latency during the BCI operations. The time-latency of 0.24 sec was measured from the trigger of FUS-mediated CBI to the appearance of rats’ tail movement. Accounting for the nerve conduction time to transmit the neural signals from the brain to the tail muscle (*i.e.*, typically less than 10 msec [33], [34]), this latency is substantially greater than the one observed during classical electric or magnetic stimulation of the cortex. This additional time delay may stem from factors such as the dependency of anesthetic states or the time needed to recruit muscle groups to elicit the tail movement. However, it also raises the interesting possibility that the fundamental mechanism for FUS-mediated neuromodulation may be different from the one governing electro-magnetic stimulation, and may introduce additional time delay during excitation. An example of such include potential involvement of astroglial systems that are sensitive to mechanical stimulation that shows slow calcium signaling [35]. Direct recordings from neuronal cells exposed to sonication, along with their cell-to-cell interactions *in vitro* or *ex vivo*, will reveal more details about the neuromodulatory effects of sonication, including the revelation of the definite causes for the discrepancy. *In vivo* evidence for the stimulatory effects of the FUS can also be evaluated by providing sonication to animals that are genetically modified to lack specific brain activities with the goal to enhance/augment their functions. Examples of such animal models can be found in genetically-engineered rodent models of neurodegenerative diseases [36].

Although the extent of the human subject’s control option in the context of BCI was limited to the “on-off” trigger that reflected the user’s intention to move the rat’s tail, expansion of control freedom can be facilitated by adopting BCI techniques that allows for characterization of spatiotemporal brain function, such as multi-channel EEG acquisition [9], [37] or real-time fMRI [6], [38]. For instance, using only surface scalp EEG electrodes, it is now possible to use neural signals related to limb kinematics for the control of complicated and analogous machine motion [39]. The adoption of such techniques will permit the detection of more diverse intentions of the operator, and subsequently will allow the operation of CBI aimed at modulating different (rodent) brain areas. For example, the imagery of each hand movement, as detected by multiple EEG montages or real-time fMRI, can be used to sonicate each of the corresponding hemispheric forepaw motor areas of the rat’s brain, resulting in mirror-like limb-to-limb control of the rodent forepaw motion. A new mode of non-invasive CBI is needed to activate the different cortical areas with specifically desired spatial and temporal accuracy, overcoming the spatial-resolution limitations of potential alternative non-invasive CBI modality, such as transcranial magnetic stimulation. A FUS technique that can generate intricate spatial patterns of the acoustic foci [32] can be especially conducive to providing simultaneous/sequential sonication to multiple areas in the brain.

The implementation discussed in this paper linked human brain signals to excite the rodent motor brain area, whereby the information flowed in only one direction due to the use of anesthetized animals. However, if both BCI and CBI are implemented between two awake human subjects, the information flow could be made bidirectional and communicative between apperceptive identities/individuals. Furthermore, neural information can be transmitted between individuals separated by a great distance using the internet protocol. Potential linking/sharing of neural processing information between individual identities can be conceptually applied to a feedback loop of the neural signal, enabling ‘autologous BBI’, which can be used to actively control/modify specific neural processing and associated cognitive/neural behaviors. Nicolelis and colleagues introduced the similar concept of ‘brain-machine-brain interface’ (BMBI) in their recent work with monkeys, whereby sensory signals, originating from the operation of BCI-actuated virtual machines, are relayed back to the brain *via* intracortical microstimulation to provide tactile feedback on the cortical level without the involvement of the peripheral nervous system [40], [41].

There are intriguing new potentials associated with the BBI, particularly relevant when used between human subjects. These potentials are implied in relation to a framework of cognitive neuroscience, coined “Neural Coupling” [42] or “Brain-to-brain coupling” [43]. The coupling refers to the phenomena in which the neural processes of one brain are coupled to the neural processes of another brain through various environmental routes, including indirect sensory/somatomotor communication. One example of such coupling is the presence of synchronous spatiotemporal patterns of brain activities that are correlated with the degree of understanding during verbal communications between a speaker and a listener [42]. The presented BBI method may be used to augment this mutual coupling of the brains, and may have a positive impact on human social behavior.

The further applicability of the FUS-based CBI to neurotherapeutics modality, as a standalone technique, as well as a part of the BBI, is both immense and far-reaching. It is reasonable to assume that further advancements and establishment of BBI between human subjects, as well as within or across species, have the potential to trigger breaking ethical questions that cannot be satisfied by applying contemporary ethical concepts. However, it is beyond the scope of this paper to address the particular moral and philosophical issues and complex challenges, possibly even undesirable consequences that may arise with the future application of this emerging technology (not necessarily within the confines of the present study). The application of BBI, therefore, will require careful consideration and resolution in the future. Certainly, the safety of the method for human use requires further scientific analysis and validation; additionally, the potential utility of such systems remains to be investigated thoroughly. Based on the successful use of commercially-available systems that allowed for the transcranial delivery of FUS applicable for humans [16], our findings suggest intriguing new possibilities for computer-assisted volitional control/communication of brain states between individuals.

## Supporting Information

Video S1 **The video recordings of BBI procedure.** A volunteer (upper left panel) signaled the intention (stimulate the motor area of a rat brain) with a thumb movement (a green dot appearing on the screen). The increased amplitude of SSVEP triggered the operation of FUS neuromodulation of a rat under the anesthesia (upper left panel), which was subsequently created the animal’s tail movement. The lower panel shows the real-time recordings of volunteer’s attention, raw SSVEP signal, SSVEP signal filtered at 15 Hz, and the tail motion (from the top to the bottom row).
(WMV)
